# Effects of trazodone on polysomnographic sleep architecture in patients with depression and sleep disorders: a systematic review and exploratory meta-analysis

**DOI:** 10.3389/fpsyt.2026.1889208

**Published:** 2026-09-10

**Authors:** Lingyan Meng, Junjun Ji, Haoran Zhang, Zhixia Cui, Yutao Yang

**Affiliations:** 1 School of Clinical Medicine, Shandong Second Medical University, Weifang, Shandong, China; 2 Weifang Mental Health Center, Weifang, Shandong, China

**Keywords:** depression, polysomnography, sleep architecture, sleep disturbance, trazodone

## Abstract

**Objective:**

Sleep disturbance and cognitive impairment are common in depression. Trazodone is often used for sleep problems and may improve cognition by modifying sleep architecture. However, its effects on specific polysomnography-derived sleep stages remain unclear. This systematic review and exploratory meta-analysis aimed to investigate trazodone’s effects on sleep architecture in patients with depression and sleep disorders.

**Methods:**

Seven databases were searched for interventional studies published up to October 2025. Random-effects models were applied to estimate pooled mean differences (MDs) and risk ratios (RRs), as appropriate.

**Results:**

Eight studies involving 980 patients were included: five randomized controlled trials and three single-arm studies. Compared with baseline, trazodone was associated with increased total sleep time and sleep efficiency, and with reduced sleep latency. Regarding sleep architecture, trazodone was linked to reduced non-rapid eye movement sleep stage 2 (N2) proportion (MD = −9.09, 95% CI −16.60 to −1.59, P = 0.017) and increased slow wave sleep (SWS) proportion (MD = 8.05, 95% CI 3.42 to 12.68, P = 0.001). No significant effects were observed for wake after sleep onset, NREM stage 1 percentage, rapid eye movement sleep percentage (REM), and REM sleep latency. Secondary outcomes suggested reduced HAMD-17 scores and higher response rates versus placebo, but more adverse events, especially somnolence and dizziness, with trazodone.

**Conclusion:**

Trazodone may improve objective sleep continuity and may be associated with changes in sleep architecture, including increased SWS and reduced N2 sleep, in patients with depression, without significantly altering REM sleep. These potential benefits should be weighed against increased adverse events.

**Systematic Review Registration:**

https://www.crd.york.ac.uk/PROSPERO/, identifier CRD420251173354.

## 1 Introduction

Depression represents a leading global public health challenge, causing substantial impairment to both physical and mental functioning. It is characterized by a high lifetime prevalence and a recurrent clinical course, with estimates ranging from 4.4% to 20% (1, 2). Depression presents with a wide range of clinical manifestations. In addition to core symptoms such as depressed mood and anhedonia, sleep disturbances and cognitive deficits, including memory impairment, are also common (3, 4). Studies have shown that between 67% and 84% of people who suffer from depression also experience sleep issues (5). In addition to subjective sleep complaints, patients with depression may also show objective abnormalities in sleep architecture on polysomnography (PSG), including shortened REM sleep latency, increased REM density, and lower proportions of N2 sleep and SWS (6–9). Because different sleep stages serve distinct roles in restorative sleep, emotional regulation, and memory consolidation (9, 10), their quantitative assessment may provide a more complete understanding of medication effects in patients with depression.

Clinically, some antidepressant drugs (such as selective serotonin reuptake inhibitors [SSRIs] and serotonin-norepinephrine reuptake inhibitors [SNRIs]) can affect sleep architecture during the early phase of treatment (11, 12). They may suppress rapid eye movement (REM) sleep, and REM rebound may occur in some patients after drug withdrawal. This may result in increased dreaming and reduced sleep quality (13). Studies have found that patients using SSRIs are at an increased risk for rapid eye movement sleep behavior disorder (RBD), a parasomnia characterized by dream-enactment behaviors and loss of normal muscle atonia during REM sleep (14–16).

Currently, benzodiazepines (BZDs) are widely used as adjunctive treatments to improve sleep in patients with depression. Although they may improve subjective sleep experience, PSG studies suggest that they can increase the proportion of non-rapid eye movement stage 2 sleep (N2), reduce slow-wave sleep (SWS), and suppress REM sleep (17). Therefore, their effects on sleep architecture may not fully reflect the physiological restoration of sleep structure. In addition, benzodiazepines may weaken the coupling between sleep spindles and slow waves (18). This coupling supports hippocampal memory reactivation and the transfer of newly learned information to the neocortex during sleep. Disruption of this process may impair sleep-dependent memory consolidation (19).

Trazodone is an antidepressant commonly used in clinical practice to improve sleep. As a triazolopyridine derivative, it exerts its pharmacological effects mainly by inhibiting the serotonin transporter (SERT) and interacting with several neurotransmitter receptors (20–22). At lower doses, antagonism of 5-HT_2_A receptors, which are involved in cortical arousal and sleep regulation, contributes to its sleep-promoting effects. Antagonism of histamine H_1_ and α_1_-adrenergic receptors, both of which are involved in maintaining arousal, further contributes to sedation (20–22). At higher doses, inhibition of SERT reduces serotonin reuptake, increases serotonergic neurotransmission, and contributes to the antidepressant effects of trazodone (20–24). Trazodone also antagonizes 5-HT_2_C receptors, which regulate serotonergic and dopaminergic neurotransmission and may further contribute to its antidepressant effects (25–27).

However, the results of previous studies on the effect of trazodone on sleep structure in patients with depression are contradictory (9, 22, 28–32). Although relevant meta-analyses have been published in recent years, most have not provided a detailed quantitative synthesis of PSG-derived sleep-stage parameters (28, 33). Therefore, it is necessary to synthesize the available evidence to characterize trazodone-related changes in sleep stages. This may help provide a more objective understanding of the PSG evidence for trazodone-related sleep improvement. Thus, we conducted a systematic review and exploratory meta-analysis to quantitatively evaluate the effects of trazodone on PSG-derived sleep parameters in patients with depression and comorbid sleep disturbance, with particular focus on sleep-stage parameters such as N1, N2, SWS, and REM sleep. Clinical efficacy and adverse events were summarized as secondary outcomes to help interpret the clinical relevance of the PSG findings.

## 2 Methods

The study protocol was registered in the International Prospective Register of Systematic Reviews (PROSPERO) (registration number: CRD420251173354). This review was reported in accordance with the PRISMA statement (34).

### 2.1 Search strategy

We searched PubMed, Web of Science, Cochrane Library, Ovid MEDLINE, Embase, Scopus, APA PsycInfo (accessed through EBSCOhost), and the World Health Organization International Clinical Trials Registry Platform (ICTRP) from database inception to October 24, 2025. A combination of controlled vocabulary and free-text terms was used. The main search terms included: Dyssomnias, sleep problems, sleep disturbances, sleep, Depression, Depressive Symptoms, and Trazodone. The complete PubMed search strategy is provided in **Supplementary Tables S1** and **S2**. The equivalent strategies were adapted for the syntax and indexing system of each database. There were no restrictions on the language of the studies. The initial search indicated that the number of randomized controlled trials (RCTs) reporting polysomnography results was relatively small. Therefore, no restrictions were placed on study design in the analysis of PSG-related sleep parameters to maximize data availability. In contrast, only RCTs were included when analyzing clinical efficacy and adverse events.

### 2.2 Eligibility criteria

Inclusion criteria were as follows (1): Patients diagnosed with depression and sleep disorders according to any of the following: Diagnostic and Statistical Manual of Mental Disorders (DSM), International Classification of Diseases (ICD), or depression treatment guidelines. Sleep disorders could also be determined through internationally validated assessment tools such as the Pittsburgh Sleep Quality Index (PSQI) or the Insomnia Severity Index (ISI). Participants were required to be adults with no gender restrictions (2). Participants in the intervention group received trazodone treatment, with no restrictions on formulation, dosage, or administration regimen. Trazodone could be used as a monotherapy or in combination with background treatments (3). In RCTs, the control group received a placebo or the same background treatment as the intervention group. For non-controlled studies, trazodone had to be the treatment under evaluation (4). Studies were required to report at least one of the following outcome categories: PSG-derived sleep parameters, clinical efficacy outcomes, or safety outcomes.

Exclusion criteria were as follows (1): non-interventional studies, such as observational studies, case reports, and reviews (2); studies for which the full text or data could not be obtained (3); duplicated publications (only the most complete report was retained) (4); studies including patients with depressive episodes of bipolar disorder.

### 2.3 Outcome measures

The primary outcomes were PSG parameters, including total sleep time (TST, min), sleep latency (SL, min), sleep efficiency (SE, %), wake after sleep onset (WASO, min), rapid eye movement sleep latency (REM-L, min), percentage of non-rapid eye movement stage 1 sleep (N1, %), percentage of N2 sleep (N2, %), percentage of SWS (SWS, %), and percentage of REM sleep (REM, %).

As previous meta-analyses have already evaluated subjective sleep outcomes, this study did not include subjective sleep scales as secondary outcomes, while clinical efficacy and adverse events were analyzed to provide clinical contextual information. The secondary outcomes included clinical efficacy and safety measures. Clinical efficacy outcomes included the 17-item Hamilton Depression Rating Scale (HAMD-17) score, responder rate (defined as the proportion of patients whose HAMD score decreased by at least 50% compared to the baseline after treatment), Clinical Global Impression-Improvement (CGI-I) responder rate (CGI-I responders were defined as patients assessed by investigators as ‘minimally improved’, ‘much improved’, or ‘very much improved’). Safety outcomes included the proportion of patients who experienced at least one adverse event (AE), the discontinuation rate due to AEs, and the incidence of specific AEs.

### 2.4 Study selection and data extraction

We imported the retrieved literature into EndNote software for management. Two researchers (Lingyan Meng and Junjun Ji) independently screened the studies, following the PRISMA guidelines (34). They first eliminated duplicate records. Then, they excluded reviews, animal studies, and irrelevant studies by reading titles and abstracts. Finally, the full texts of the remaining studies were assessed for eligibility, and reasons for exclusion were documented. In cases of disagreement, a third investigator was involved. A consensus was reached through discussion among the three authors.

Data extraction included (1) general information: the first author, publication year, study country, study design, and sample size (2); sample demographics: age, % females, and diagnostic criteria (3); intervention characteristics: trazodone formulation, dosage, and duration of trazodone (4); study results: PSG parameters, HAMD-17 score, responder rate, CGI-I responder rate, proportion of patients with at least one AE, discontinuation rate due to AEs, and incidence of specific AEs. If data were missing, we contacted the corresponding authors by email to obtain the data and detailed information.

### 2.5 Quality assessment

Two authors (Lingyan Meng and Junjun Ji) independently evaluated the quality of studies according to the guidelines of the Cochrane Handbook for Systematic Reviews of Interventions (35). For RCTs, the revised Cochrane risk-of-bias tool (RoB 2) was used to assess the risk of bias across five domains, rated as “low risk”, “some concerns”, or “high risk” (36). Non-randomized studies of interventions were assessed using the ROBINS-I tool, which evaluates bias across seven domains: confounding, participant selection, classification of interventions, deviations from intended interventions, missing data, outcome measurement, and selection of the reported result. Each domain was rated as “low”, “moderate”, “serious”, or “critical” risk to determine the overall risk of bias for each study (37). Disagreements were resolved through discussion.

### 2.6 Data analysis

All analyses were performed using RevMan 5.4 and Stata 15 (StataCorp, College Station, TX, USA). To maximize the utility of limited PSG evidence, a single-group pre-post meta-analysis was conducted for sleep parameters. For RCTs, only data from the trazodone group at baseline and endpoint were extracted. For single-arm trials, baseline and post-treatment data were extracted. When the standard deviation (SD) of change scores was missing, we estimated these values by assuming a correlation coefficient (r) of 0.5. To ensure the robustness of the findings, we also performed sensitivity analyses using r values of 0.2 and 0.8. Pooled effect sizes reflected the changes from baseline to post-treatment within trazodone-treated participants and were interpreted as exploratory within-group estimates. To analyze clinical efficacy and safety, we extracted endpoint data from both the intervention and control groups of the included RCTs for between-group comparison.

Continuous variables were expressed as mean differences (MDs) with 95% confidence intervals (CIs). Dichotomous variables were assessed using risk ratios (RRs) and 95% CIs. A two-sided P value of less than 0.05 was considered statistically significant. Heterogeneity was evaluated using the I^2^ statistic (37). A random-effects model was primarily employed for the meta-analysis. A leave-one-out sensitivity analysis was performed by sequentially excluding one study at a time and recalculating the pooled estimate to assess the robustness of the overall results and identify potential sources of heterogeneity. Because fewer than 10 studies were included for each outcome, Egger’s and Begg’s tests were not performed to assess publication bias (36, 38).

## 3 Results

### 3.1 Search results

**Figure 1:**
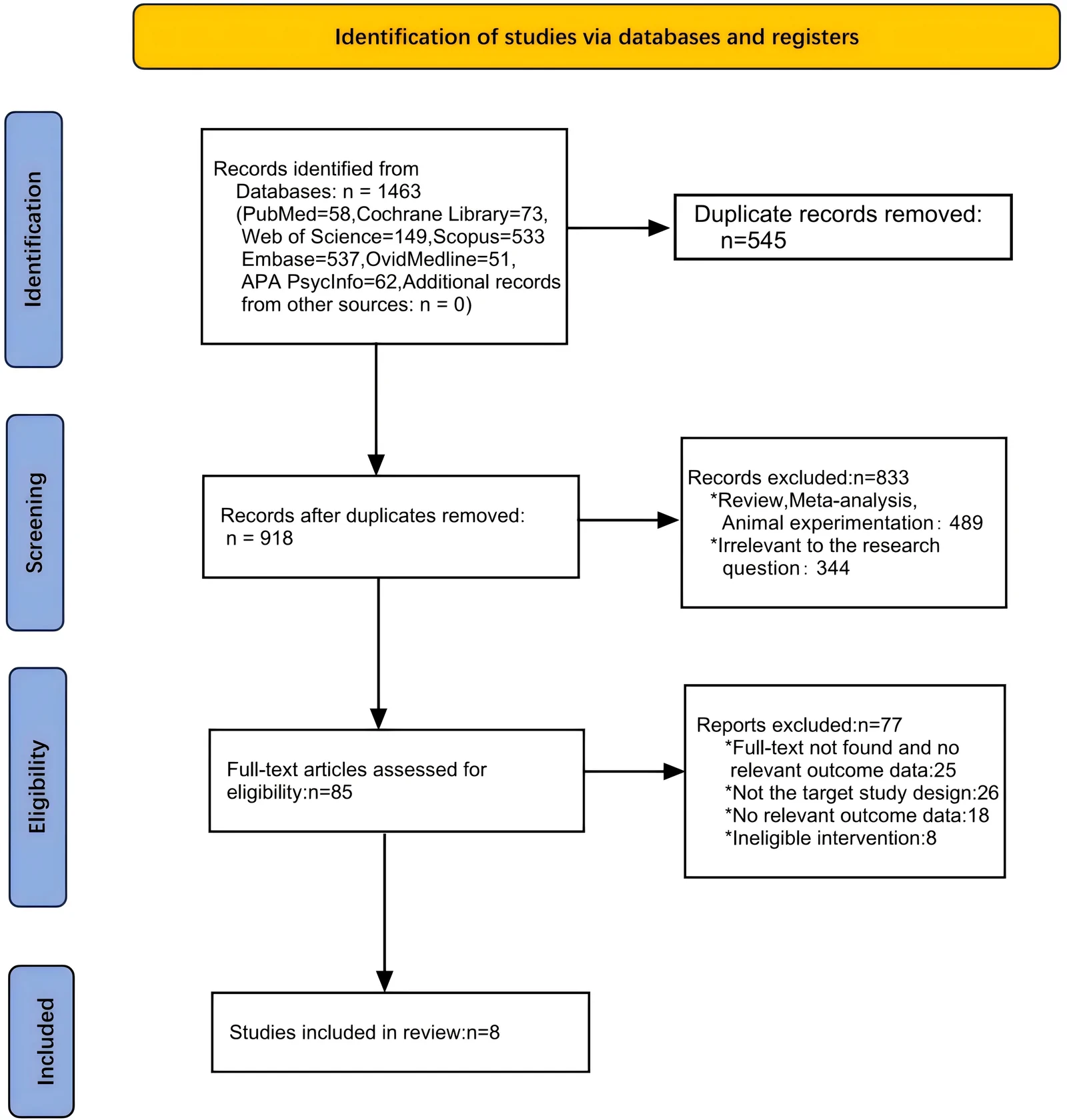
Flow diagram indicating the process of selecting studies for the review.

The initial search identified 1,463 articles, including PubMed (n = 58), Cochrane Library (n = 73), Web of Science (n = 149), Scopus (n = 533), Embase (n = 537), Ovid MEDLINE (n = 51), and APA PsycInfo (n = 62). After exclusion of 545 duplicate records, two researchers independently screened the titles and abstracts of the remaining 918 articles. According to the inclusion and exclusion criteria, 85 articles were evaluated in full text, and 8 studies were ultimately included (39–46). The study selection process is shown in **Figure 1**.

### 3.2 Characteristics of included studies

A total of 8 studies were included. The studies were published between 1992 and 2025. In terms of geographical distribution, most studies were conducted in Europe (62.5%) (39, 41, 42, 45, 46), followed by North America (25%) (43, 44) and Asia (12.5%) (40). Study designs consisted of three parallel-group RCTs (40, 43, 44), two crossover RCTs (39, 41), and three single-group pre-post studies (42, 45, 46). The total sample size was 980, with a range of 6 to 406 (mean = 123). Among the five studies reporting PSG outcomes, participant ages ranged from 20 to 59 years (39, 41, 42, 45, 46), whereas the remaining three studies did not report actual age ranges (40, 43, 44). The mean age of participants across all the included studies was 40.8 years, and approximately 69.2% were women. All studies used internationally recognized diagnostic criteria (such as DSM, ICD) to diagnose depression. The daily dose of trazodone ranged from 50 to 400 mg, and treatment duration ranged from 1 day to 6 weeks (mean = 4.4 weeks). Four of these studies had a 6-week course (40, 43, 44, 46), two had a 5-week course (42, 45), one had a 1-week course (39), and one had a 1-day course (41). There was concomitant medication use in two studies (39, 41). One study reported stable antidepressant treatment without further specification (41), whereas the other specifically reported stable SSRI treatment (39). Regarding outcome measures, three studies reported only clinical efficacy and safety data (40, 43, 44). Five studies reported PSG data (39, 41, 42, 45, 46). Two of these included clinical data (39, 41). All five studies mentioned adaptation nights, during which participants slept in the laboratory before formal PSG recording to become familiar with the environment and monitoring equipment and to reduce first-night effects on sleep architecture (47). The characteristics of all included trials are shown in **Table 1**; **Supplementary Table S3**.

**Table 1:** Characteristics of the eight included studies.

First author	Year	Sample size	Female%	Type of Study	Intervention (Experimental/ Control)	Dosage	Duration	Outcome measures
Bavato (41)	2025	23	69.57	single-center, Randomized, double-blind, placebo-and active comparator-controlled, crossover trial	Experimental: Trazodone /Control: Placebo	1.5mg/kg (72–156 mg)	one night	PSG (TST, SL, WASO, N1(%), N2(%), REM (%), SE), ≥1AE, Dizziness, Somnolence, Nausea
Zhang (40)	2014	183 (Experimental)/ 180 (Control)	61.43	Multicenter, randomized, double-blind, placebo-controlled trial	Experimental: Prolonged-release trazodone/Control: Placebo	150–450 mg/day	six weeks	HAMD, Response rate, CGI-Iresponse rate, ≥1 AE, Dizziness, Dry mouth, Somnolence, Nausea
Sheehan (43)	2009	202 (Experimental)/ 204 (Control)	64.04	Multicenter, randomized, double-blind, placebo-controlled trial	Experimental: Extended-release trazodone/Control: Placebo	150–375 mg/day	six weeks	HAMD, Response rate, CGI-I response rate, ≥1 AE, the discontinuation rate due to AEs, Dizziness, Dry mouth, Somnolence, Nausea
Kaynak (39)	2004	12	100	randomized, double-blind, placebo-controlled, Crossover trial	Experimental: Trazodone/Control: Placebo	100 mg/day	one week	HAMD, PSG (TST, SL, SE, N1(%), N2(%), SWS (%), REM (%), REM-L), ≥1 AE
Arriaga (45)	1997	9	100	Single-blind, within-subject, placebo-controlled trial	Trazodone	50-250 mg/day	five weeks	PSG (TST, SE, SL, REM-L, N1(%), N2(%), SWS (%), REM (%))
Parrino (46)	1994	6	50	Single-blind, within-subject, placebo-controlled trial	trazodone controlled release	50-150 mg/day	six weeks	PSG (TST, SE, SL, REM-L, WASO, N1(%), N2(%), SWS (%), REM (%))
Cunningham (44)	1994	77 (Experimental)/ 76 (Control)	33.3	Multicenter, randomized, double-blind, placebo-controlled trial	Experimental: Trazodone/Control: Placebo	150–400 mg/day	six weeks	the discontinuation rate due to AEs, Dizziness, Dry mouth, Somnolence, Nausea
van Bemmel (42)	1992	8	75	Single-blind, within-subject, placebo-controlled trial	Trazodone	100–400 mg/day	five weeks	PSG (TST, SE, SL, REM-L, WASO, N1(%), N2(%), SWS (%), REM (%))

≥1 AE, proportion of patients who experienced at least one adverse event; CGI-I responders, patients assessed by investigators as “minimally improved”, “much improved”, or “very much improved” on the Clinical Global Impression-Improvement scale; DSM, Diagnostic and Statistical Manual of Mental Disorders; HAMD, Hamilton Depression Scale; N1, non-rapid eye movement stage 1; N2, non-rapid eye movement stage 2; NR, not reported; PSG, polysomnography; REM, rapid eye movement; REM-L, latency to rapid eye movement sleep; Response rate, percentage of patients achieving a ≥50% reduction in the total HAMD-17 score from baseline to the end of the study; SE, sleep efficiency index; SL, sleep latency; SSRIs, selective serotonin reuptake inhibitors; SWS, slow-wave sleep; TST, total sleep time; WASO, waking time after sleep onset.

### 3.3 Quality assessment

**Figure 2:**
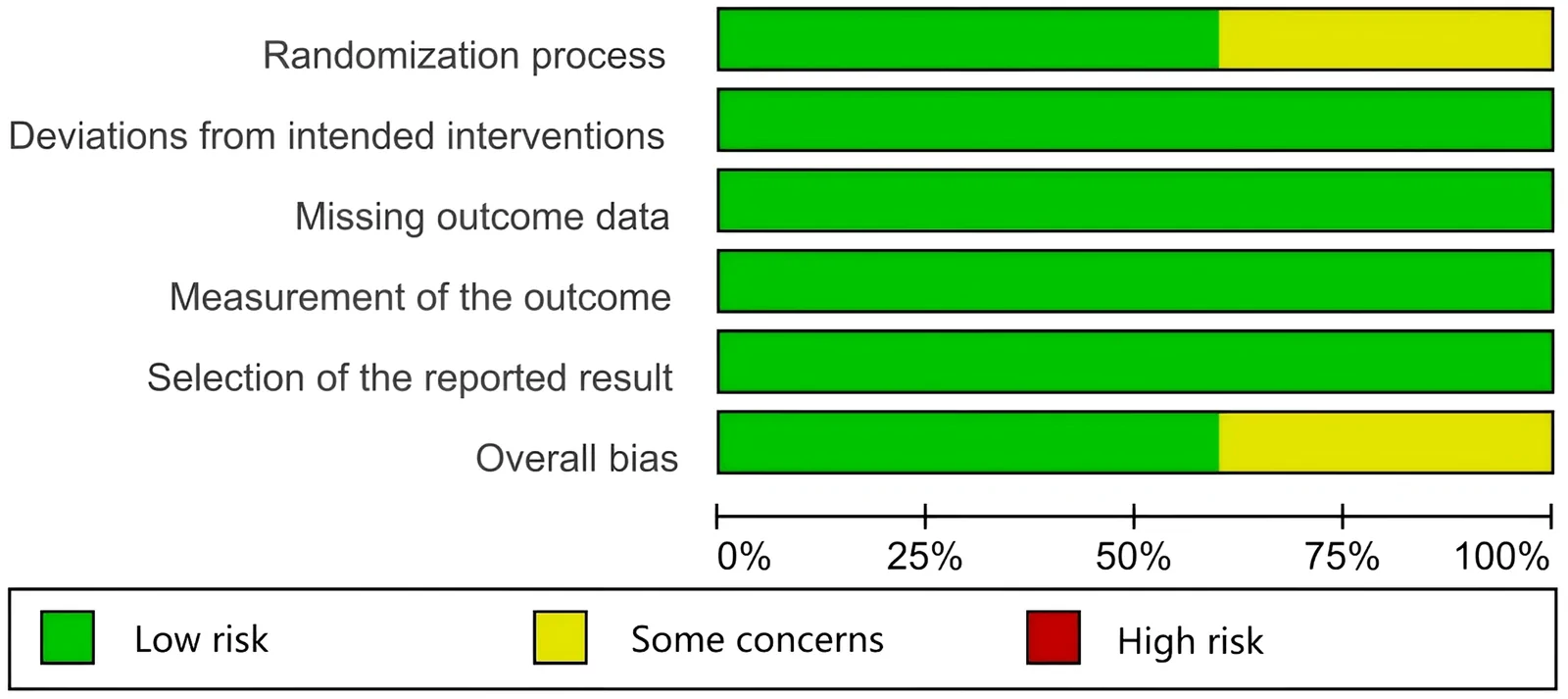
Risk of bias graph for RoB 2 quality assessment.

**Figure 3:**
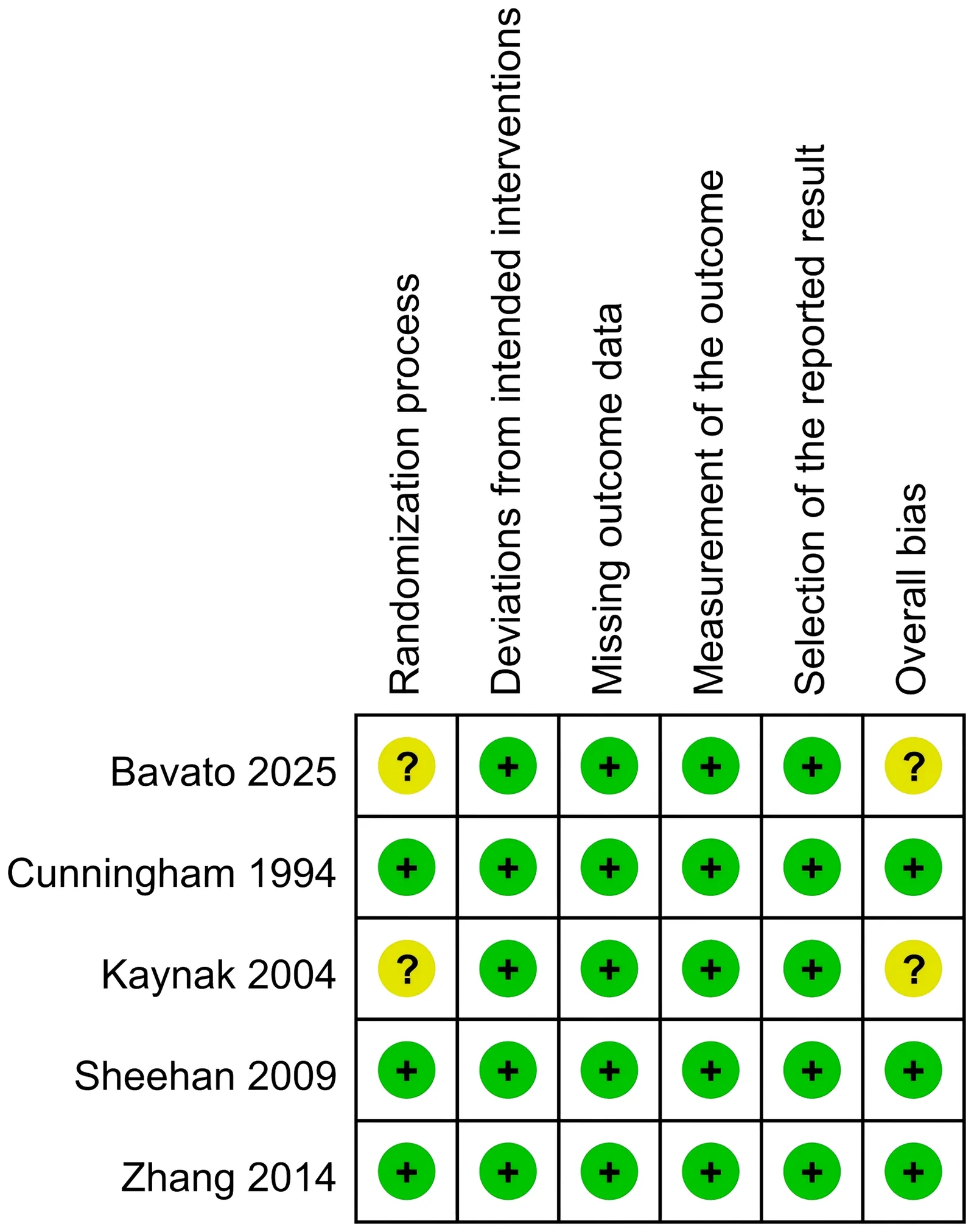
Risk of bias summary for RoB 2 quality assessment.

The Cochrane RoB 2 tool was used to assess the risk of bias in RCTs (36). All RCTs had a double-blind design with detailed reporting of attrition and relatively objective PSG results. Therefore, they were rated as “low risk” in the domains of “deviation from intended intervention”, “selection of the reported result”, and “measurement of the outcome”. Two studies were rated as “some concerns” in the domain of “randomization process” due to an unclear description of baseline comparability (39, 41). The remaining three studies were rated as “low risk” (40, 43, 44). The studies by Bavato et al. and Kaynak et al. (39, 41) were rated as “some concerns” for overall risk of bias, while the remaining three were rated as “low risk” (**Figures 2**, **3**).

Single-arm trials (42, 45, 46) were evaluated using the ROBINS-I tool (37). All studies were rated as “serious” in the “confounding” domain due to the lack of a control group. Two studies did not clearly limit combined medication (42, 45), and one study had unclear blinding of outcome assessment (42), which was rated as “moderate” in those fields. Overall, due to the limitations of study design, the overall risk of bias of all included single-arm trials was “serious” (**Table 2**).

**Table 2:** Risk of bias for the 3 included publications, based on the ROBINS-I tool (low, moderate, serious, critical).

Author, year	Type of bias							Overall rating
	Bias due to confounding	Bias in selection of participants into the study	Bias in classi-fication of interventions	Bias due to deviations from intended interventions	Bias due to missing data	Bias in measurement of outcomes	Bias in selection of the reported result	
Arriaga, 1997 (45)	Serious	Low	Low	Moderate	Low	Low	Low	Serious
Parrino, 1994 (46)	Serious	Low	Low	Low	Low	Low	Low	Serious
Van Bemmel,1992 (42)	Serious	Low	Low	Moderate	Low	Moderate	Low	Serious

ROBINS-I tool, the risk of bias in non-randomized studies of interventions tool.

### 3.4 Primary efficacy outcomes

**Figure 4:**
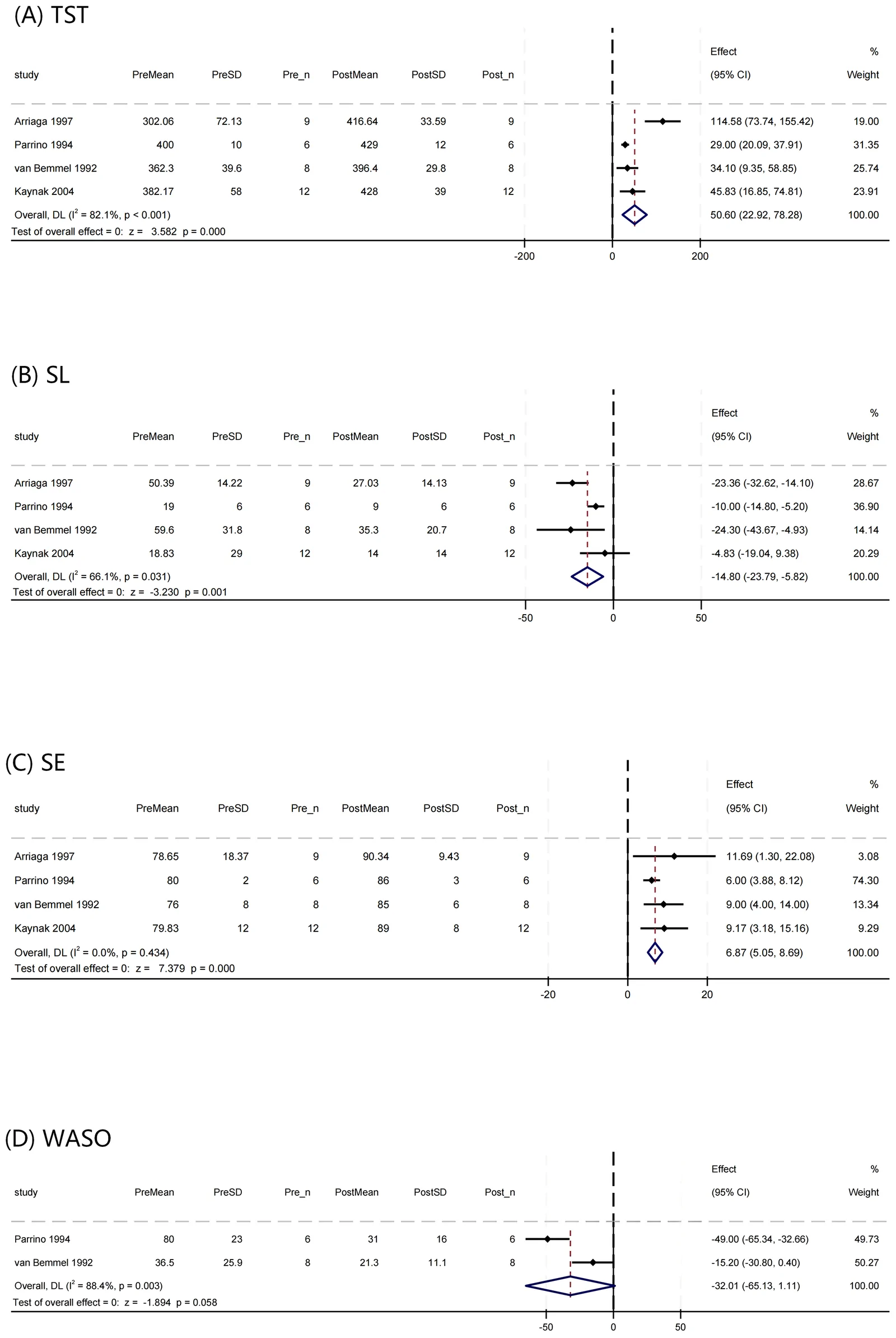
Forest plots of the meta-analysis for sleep continuity: **(A)** TST, total sleep time, **(B)** SL, sleep latency, **(C)** SE, sleep efficiency, **(D)** WASO, wake after sleep onset.

Four studies reported data on sleep parameters and were included in the quantitative analysis, including one RCT (39) and three single-group pre-post studies (42, 45, 46). In the exploratory within-group analyses, trazodone treatment was associated with improvements in objective sleep continuity compared with baseline. Specifically, trazodone treatment was associated with increased TST (MD = 50.598, 95% CI 22.915 to 78.281, P = 0.000) and SE (MD = 6.870, 95% CI 5.045 to 8.695, P = 0.000) compared with baseline, while shortening SL (MD = -14.803, 95% CI -23.787 to -5.820, P = 0.001) (**Figure 4**).

**Figure 5:**
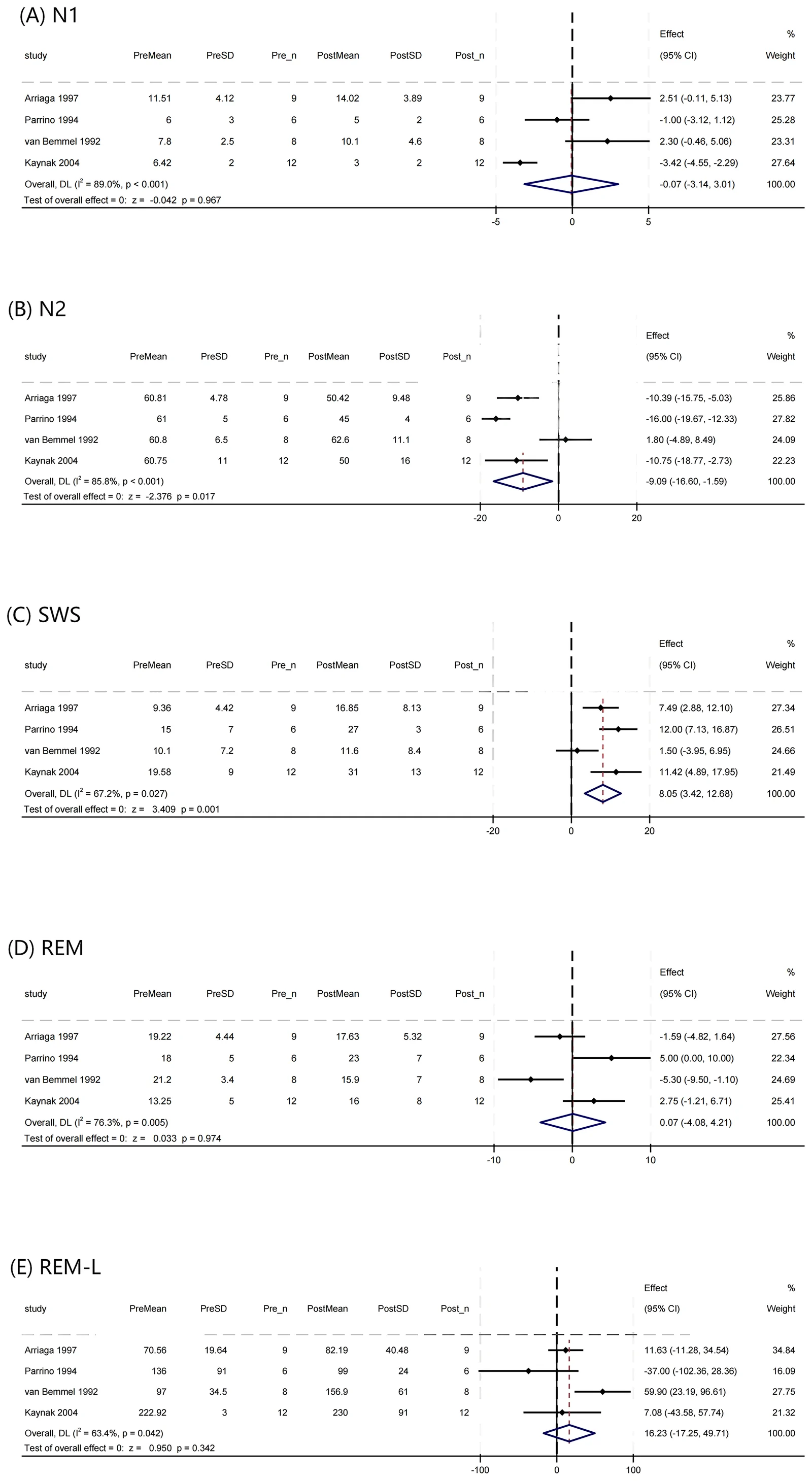
Forest plots of the meta-analysis for sleep stage distribution: **(A)** N1, non-rapid eye movement stage 1 sleep, **(B)** N2, non-rapid eye movement stage 2 sleep, **(C)** SWS, slow-wave sleep, **(D)** REM, rapid eye movement sleep, **(E)** REM-L, rapid eye movement sleep latency.

Two single-arm studies (42, 46) provided WASO data, which showed a decreasing trend from baseline (MD = -32.009, 95% CI -65.131 to 1.114, P = 0.058) (**Figure 4**). The difference was not statistically significant. There was also no statistically significant change in REM-L (MD = 16.226, 95% CI -17.254 to 49.706, P = 0.342) (**Figure 5**).

Regarding sleep stages, reductions in the proportion of N2 sleep (MD = -9.095, 95% CI -16.596 to -1.593, P = 0.017) and increases in the proportion of SWS were observed after trazodone treatment compared with baseline (MD = 8.053, 95% CI 3.423 to 12.682, P = 0.001). However, changes in N1 (MD = -0.065, 95% CI -3.142 to 3.012, P = 0.967) and REM sleep proportion (MD = 0.069, 95% CI -4.076 to 4.214, P = 0.974) were not statistically significant (**Figure 5**).

We performed sensitivity analyses by setting correlation coefficients (r) of 0.2 and 0.8. The results were consistent with the main analyses. This suggests that the results were robust to the setting of r (**Supplementary Table S4**).

### 3.5 Additional analyses for primary efficacy outcomes

#### 3.5.1 Sensitivity analyses

We found that the heterogeneity of most sleep parameter indicators was relatively high. Therefore, a leave-one-out sensitivity analysis was conducted. The results showed that the study by Arriaga et al. (45) was the main source of heterogeneity for TST and SL, while the study by van Bemmel et al. (42) was the main source of heterogeneity for SWS and REM-L. The heterogeneity significantly decreased after their exclusion. Specifically, compared with the baseline, the TST of patients after trazodone treatment increased (I² = 0%, MD = 30.840, 95% CI 22.786 to 38.893, P = 0.000), SL decreased (I² = 22.8%, MD = -10.611, 95% CI -17.444 to -3.778, P = 0.002), and the percentage of SWS increased (I² = 0.0%, MD = 9.995, 95% CI 7.018 to 12.972, P = 0.000). The effect of REM-L (I² = 0.0%, MD = 6.429, 95% CI -13.454 to 26.312, P = 0.526) did not reach statistical significance (**Supplementary Table S5**). The sensitivity analysis showed that the overall effect direction of most sleep indicators remained stable after removing any single study. This suggested that the overall findings were mainly robust. However, for N2, we found that the heterogeneity significantly decreased and the pooled effect size increased after removing van Bemmel et al. (42). Conversely, removing other studies altered the statistical significance of the results. Therefore, the results of N2 should be interpreted with caution (**Supplementary Table S6**).

Since the number of studies included for each outcome indicator was less than 10, publication bias tests were not formally assessed.

#### 3.5.2 RCT-only analyses

As a supplementary analysis, we performed a between-group pooled analysis restricted to RCTs. Two RCTs (39, 41) reported PSG parameters and indicated higher TST (MD = 26.68, 95% CI 5.03 to 48.33, P = 0.02), improved SE (MD = 6.09, 95% CI 1.42 to 10.75, P = 0.01), and reduced WASO (MD = -19.52, 95% CI -29.95 to -9.09, P = 0.0002) with trazodone treatment. There was no significant effect on SL (MD = 0.13, 95% CI -8.89 to 9.15, P = 0.98) or sleep stages. The specific data were as follows: N1 (%) (MD = -1.39, 95% CI -6.77 to 4.00, P = 0.61), N2 (%) (MD = -3.92, 95% CI -9.81 to 1.98, P = 0.19), SWS (%) (MD = 7.22, 95% CI -0.93 to 15.38, P = 0.08), and REM (%) (MD = -1.88, 95% CI -5.40 to 1.54, P = 0.29) (**Supplementary Table S7**).

### 3.6 Clinical symptom improvement

Three RCTs (40, 43, 44) reported HAMD-17 scores for trazodone and placebo groups. The results showed that patients who received trazodone had a statistically greater improvement in symptoms than those who received placebo. Two RCTs (40, 43) evaluated response rates and CGI-I response rates, which were higher in the trazodone group than in the control group (**Table 3**).

**Table 3:** Meta-analysis results for secondary outcomes.

Outcomes	Number of studies	Heterogeneity	RR	95%CI	Z	P
Clinical efficacy						
Response rate	2	P=0.20; I^2^ = 39.1%	1.44	1.24, 1.68	4.70	0.00001
CGI-I responder	2	P=0.77; I^2^ = 0.0%	1.10	1.02, 1.18	2.52	0.01
Safety assessment						
≥1 AE (n)	4	P<0.0001;I^2^ = 88.4%	1.66	0.89, 3.08	1.60	0.11
Discontinuation due to AEs (n)	2	P=0.65; I^2^ = 0.0%	4.78	2.38, 9.62	4.39	<0.0001
Dizziness	4	P=0.12; I^2^ = 48.8%	2.80	2.02, 3.88	6.16	<0.00001
Dry mouth	3	P=0.21; I^2^ = 35.6%	2.09	1.49, 2.91	4.32	<0.0001
Somnolence	4	P=0.02; I^2^ = 69.8%	3.82	1.68, 8.71	3.19	0.001
Nausea	4	P=0.37; I^2^ = 5%	2.04	1.40, 2.98	3.71	0.0002

CI, confidence interval; I², inconsistency statistic; RR, risk ratio.

### 3.7 Safety and adverse events

With respect to tolerability and adverse events, there was no significant difference in the proportion of patients who experienced at least one AE between the trazodone group and the placebo group. The discontinuation rate due to AEs was significantly higher in the trazodone group than in the placebo group. The incidences of dizziness, dry mouth, somnolence, and nausea were all significantly higher in the trazodone group than in the control group (**Table 3**). Sensitivity analyses of these adverse reactions indicated that the study by Sheehan et al. (43) was the main source of heterogeneity in the proportion of at least one AE and the incidence of somnolence. When this study was excluded, the heterogeneity of both indicators decreased (the proportion of at least one AE: I^2^ = 43.9%, RR = 1.92, 95% CI 1.49 to 2.48, P < 0.00001; the incidence of somnolence: I^2^ = 0%, RR = 5.86, 95% CI 3.29 to 10.45, P < 0.00001).

## 4 Discussion

Clinically, sleep disturbance in depression may present as insomnia or hypersomnolence (48). Insomnia is more common in melancholic depression and is often characterized on polysomnography by impaired sleep continuity, reduced slow-wave sleep (SWS), shortened REM sleep latency, and increased REM density. Hypersomnolence is more often seen in atypical depression. It is generally associated with longer total sleep time and less pronounced abnormalities in REM sleep and SWS (9). Evidence also suggests a bidirectional relationship between depression and sleep disturbance (49). In patients with depression, insomnia is associated with greater symptom severity, poorer psychosocial function, and lower quality of life (50). Hypersomnolence is also linked to functional impairment, failure to achieve remission, and poorer clinical outcomes (50, 51). Guerrera et al. found that disruption of biological rhythms may partly mediate the relationship between depressive symptoms and impaired psychosocial functioning, with sleep disturbance representing an important component of this disruption (52). In addition, SWS and REM sleep play important roles in memory consolidation and emotional regulation (9, 10). Taken together, these findings suggest that improving sleep continuity and sleep architecture may not only reduce nighttime symptoms but may also support broader recovery, with potential benefits for depressive symptoms and cognitive functioning.

This systematic review and exploratory meta-analysis suggested that trazodone treatment was associated with improved objective sleep continuity in patients, mainly manifested as increases in TST and SE, and a trend of shortening SL was also observed. These findings are consistent with previous studies (13, 53–55). Regarding sleep stages, previous studies indicated that trazodone may increase SWS, but its effect on REM sleep was unclear (12, 53). The results of this study showed that trazodone treatment appeared to increase the percentage of SWS and decrease the percentage of N2 sleep, whereas no statistically significant changes were observed in the percentage of REM sleep or REM latency.

The impact of trazodone on sleep architecture may differ from that of other antidepressants. In clinical practice, some antidepressants with activating properties, including SSRIs and SNRIs, have been reported to affect patients’ sleep, such as prolonging sleep latency, reducing sleep efficiency, and disrupting sleep continuity (11–13). These effects may contribute to insomnia or agitation, particularly during the early phase of treatment (11). In contrast, this study found that trazodone was associated with improved sleep continuity. During the early treatment period, when antidepressant effects may not yet be fully established (56, 57), improvement in sleep continuity may contribute to better subjective sleep perception and may potentially facilitate treatment adherence (58, 59). Additionally, some antidepressants may suppress REM sleep and prolong its latency (11). After discontinuation, REM rebound can occur, affecting sleep quality by increasing the frequency of dreaming (13). Evidence suggests that SSRIs may increase the risk of RBD in patients (14, 15). Previous studies have also shown that REM sleep is involved in the processing of negative emotional memories. Persistent inhibition of REM sleep may interfere with the processing of emotional memories in patients and may be associated with emotional blunting (60, 61). In this review, the percentage of REM sleep did not change significantly after trazodone treatment, suggesting that its effects on REM sleep may differ from those of some SSRIs and SNRIs. However, because related studies remain limited, whether these differences translate into clinical benefits requires further investigation.

BZDs are commonly used sedative-hypnotic drugs and are often combined with SSRIs to improve sleep in patients with depression (18). They can shorten sleep latency, reduce WASO, and improve sleep continuity. However, they can also increase the proportion of N2 and reduce the SWS and REM sleep (17). In contrast, the present study showed that trazodone treatment was associated with a decreased proportion of N2 sleep and an increased proportion of SWS, suggesting a relative shift from lighter N2 sleep toward deeper SWS rather than only sedation or prolonged sleep duration. The increase in SWS observed with trazodone may represent a sleep pattern more consistent with restorative sleep and may potentially contribute to improvements in depressive symptoms (62, 63). However, in patients with hypersomnolence, its sedative effects may increase daytime sleepiness (11). Clinical use should therefore be individualized according to the patient’s sleep profile.

Trazodone has dose-dependent pharmacological effects, and different dose ranges are generally used for different clinical purposes. Doses of 25–150 mg/day are more often used for insomnia or sleep-promoting effects, whereas doses of 150–400 mg/day are mainly used for antidepressant treatment, although sleep may also be affected at these higher doses (64). However, these clinical dose ranges should not be regarded as a clear threshold for changes in sleep architecture. It is worth noting that the leave-one-out sensitivity analysis for the N2 sleep stage indicated that the study by van Bemmel et al. was the primary source of the observed high heterogeneity (42). When this study was excluded, the statistical heterogeneity decreased, and the overall effect size seemed larger, remaining statistically significant. However, if any other study was excluded alone, the combined result would no longer be statistically significant. This suggests that the findings for the N2 sleep stage were particularly influenced by van Bemmel et al.’s study. This difference may be related to the dose of trazodone. In the study by van Bemmel et al., trazodone was administered at a maintenance dose of 300 mg/day, which was the highest maintenance dose among the five studies reporting PSG outcomes. These observations raise the possibility that the change in the N2 sleep stage proportion may be partly related to trazodone dose.

Beyond the findings for N2, descriptive patterns across individual studies suggested that trazodone dose might partly contribute to differences in other sleep parameters. In van Bemmel’s study, trazodone was associated with REM sleep suppression and had no significant effect on SWS, which differed from the findings of the other three studies (39, 45, 46). Although Arriaga et al. also used a relatively high maintenance dose (250 mg daily) (45), their results were closer to those of Parrino et al.’s study (46). In Parrino et al.’s study, the trazodone dose was lower, and no specific effect on REM sleep was observed, but the proportion of SWS increased. This pattern raises the possibility that the relatively high maintenance dose of 300 mg/day used in van Bemmel et al.’s study may have partly contributed to the distinct changes in sleep structure. Mechanistically, previous studies have suggested that activation of the 5-HT_1_B receptor can shorten REM sleep duration (65). At higher doses, trazodone may increase the concentration of serotonin in the synaptic cleft by inhibiting the SERT, thereby potentially activating 5-HT_1_B receptors (21). This might provide one possible explanation for the REM sleep suppression observed in van Bemmel et al.’s study. Additionally, trazodone has dose-dependent effects on 5-HT_2_A/_2_C, H_1_, and α_1_-adrenergic receptors at different doses (21). This may also contribute to differences in sleep-stage distribution across studies. Nevertheless, given the small number of studies, subgroup analysis could not be performed to further determine a specific dose threshold for changes in sleep architecture.

Across the included studies, changes in the proportions of N1 and N2 stages of sleep were seen alongside changes in SWS and REM sleep. In some studies (39, 45, 46), increases in SWS were observed together with decreases in the proportion of N1 or N2 stages. In another study (42), a decrease in REM sleep was accompanied by an increase in the proportion of N2 sleep. A possible explanation is that trazodone may primarily affect SWS and REM sleep, with corresponding changes in the relative proportions of N1 and N2 stages.

Furthermore, in studies involving only female participants (39, 45), improvement in sleep continuity appeared more pronounced. This pattern raises the possibility that sex may influence the effects of trazodone on sleep parameters. However, due to the limited number of existing studies, reliable quantitative analyses based on dose or sex subgroups could not be conducted. Therefore, these observations should be interpreted cautiously and require further verification in future studies.

When the meta-analysis was restricted to RCTs (39, 41), no statistically significant effects of trazodone on sleep-stage parameters were observed. This finding may partly reflect limited statistical power due to the small number of RCTs. Additionally, the treatment periods in the two studies were relatively short, lasting 1 day and 1 week, respectively. This might not have been sufficient to comprehensively evaluate the impact of trazodone on sleep architecture. It should be particularly noted that the change in WASO approached statistical significance (P = 0.058) in the pooled analysis, but when the analysis was limited to RCTs, the change in effect size was statistically significant. However, given the limited number of RCTs and the short follow-up periods, these results should be interpreted with caution.

In terms of clinical efficacy, the findings of this systematic review and meta-analysis suggest that trazodone may alleviate the severity of symptoms in patients with depression. Compared with placebo, trazodone was associated with reductions in HAMD-17 scores, an increased responder rate, and a higher CGI-I responder rate. Regarding safety and tolerability, the proportion of patients in the trazodone group experiencing at least one adverse event and the treatment discontinuation rate due to adverse events were both higher than those in the placebo group. Specifically, compared with placebo, patients taking trazodone were more prone to somnolence and dizziness, which may be related to its H_1_ histamine receptor antagonism and α_1_-adrenergic receptor antagonism. Therefore, in clinical practice, clinicians should consider advising patients to avoid driving after taking trazodone and to be vigilant about the risk of falls due to orthostatic hypotension.

This study has the following advantages: Previous reviews on the effects of trazodone on sleep in patients with depression were mostly narrative (9, 22, 30). Recent meta-analyses mainly focused on clinical efficacy and safety outcomes (28, 33). Quantitative evidence on trazodone-associated changes in PSG-derived sleep architecture, especially stage-specific parameters such as N1, N2, SWS, and REM sleep, remains limited. To fill this gap, this study quantitatively synthesized PSG-derived sleep parameters as the primary outcomes, while clinical efficacy and adverse events were analyzed as secondary outcomes to provide clinical context. By examining specific sleep stages, this study may provide a more comprehensive understanding of trazodone-related changes in sleep architecture and may inform future research exploring whether sleep architecture alterations contribute to cognitive and clinical outcomes in patients with depression. In addition, to improve measurement consistency and reduce subjective assessment bias, this study included only sleep outcome measures assessed via PSG.

This study has several limitations. First, this meta-analysis included both RCTs and single-arm trials in the assessment of sleep structure parameters. Although the included RCTs were of relatively high quality, single-arm trials lack control groups and therefore have inherent limitations in controlling for confounding factors and internal validity. Consequently, causal interpretation of the pooled PSG findings is limited. Second, the number of included studies was small, and the overall sample size was limited. This may have reduced the statistical power and robustness of the results. The results should be interpreted with caution. Third, participants in two of the included studies remained on stable antidepressant treatment. Concomitant antidepressant use may have affected sleep architecture and made it more difficult to isolate the effects of trazodone. Fourth, several PSG analyses were based on pre-post changes. The assumed correlation coefficients used for variance estimation may have influenced the pooled estimates, although sensitivity analyses were performed. Fifth, this review focused on objective PSG-derived sleep parameters and did not quantitatively synthesize subjective insomnia outcomes such as PSQI or ISI. Therefore, the findings mainly reflect objective sleep architecture and continuity rather than subjective sleep improvement. Sixth, treatment duration varied considerably across studies, ranging from one day to six weeks. Outcomes assessed after the first dose may primarily reflect the acute sedative effects of trazodone on sleep. In contrast, outcomes assessed after several weeks may reflect the combined effects of continued treatment and improvement in depressive symptoms on sleep architecture (64). Differences in treatment duration may therefore have contributed to variation in the magnitude or pattern of the observed sleep effects and limited the assessment of the long-term effects of trazodone on sleep architecture. Seventh, cognitive outcomes were not directly evaluated in the included studies. Therefore, although specific sleep stages play important roles in memory consolidation and emotional regulation, the possible cognitive implications of trazodone-related changes in sleep architecture remain speculative. Finally, potential dose- or sex-related patterns in sleep architecture were based on descriptive observations across a small number of studies and should be regarded as exploratory. In the future, large-scale, high-quality RCTs about PSG sleep parameters are needed to further characterize the effects of trazodone on specific sleep stages.

## 5 Conclusion

This systematic review and exploratory meta-analysis indicates that trazodone treatment may improve sleep continuity in patients with depression and sleep disorders. Specifically, it is reflected by an increase in TST and SE, along with a trend toward reduced SL. In terms of sleep architecture, trazodone may be associated with an increase in the proportion of SWS and a reduction in N2 sleep. Meanwhile, there were no significant changes in the proportion of REM sleep and REM latency. Limited data suggest that dosing regimens and sex may affect its impact on sleep architecture, but further verification is needed. By quantitatively examining specific sleep stages, this study might provide preliminary information for understanding trazodone-related changes in sleep architecture and for exploring the potential role of sleep in mediating cognitive benefits. In terms of clinical outcomes, trazodone was associated with greater improvements in depressive symptoms than placebo. Regarding safety, its use was linked to adverse events in the central nervous system (such as somnolence and dizziness) and the gastrointestinal tract (such as nausea). Due to the limited number of included studies and the quality of evidence, these results should be interpreted with caution. Future high-quality RCTs with larger sample sizes and PSG-derived sleep parameters as primary outcomes are needed to further clarify the comprehensive therapeutic value of trazodone in patients with depression and sleep disorders.

## Data Availability

The original contributions presented in the study are included in the article/**Supplementary Material**. Further inquiries can be directed to the corresponding author.
