# Supplementary material for: Effects of trazodone on polysomnographic sleep architecture in patients with depression and sleep disorders: a systematic review and exploratory meta-analysis
Source: Front Psychiatry. 2026 Sep 10;17:1889208. doi: 10.3389/fpsyt.2026.1889208 (PMC13601357; doi:10.3389/fpsyt.2026.1889208)
Supplement: Supplementary file 1 [file Supplementaryfile1.docx]

**Supplementary Material**

Supplementary Table S1. Detailed PubMed search strategy

| **Search number** | **Query** |
| --- | --- |
| 1 | "Dyssomnias"[Mesh] |
| 2 | sleep problems[Title/Abstract] OR sleep disturbances[Title/Abstract] OR sleep disorders[Title/Abstract] OR Dyssomnia[Title/Abstract] OR Sleep Disorders, Extrinsic[Title/Abstract] OR Extrinsic Sleep Disorder[Title/Abstract] OR Extrinsic Sleep Disorders[Title/Abstract] OR Sleep Disorder, Extrinsic[Title/Abstract] OR Adjustment Sleep Disorder[Title/Abstract] OR Adjustment Sleep Disorders[Title/Abstract] OR Sleep Disorders, Adjustment[Title/Abstract] OR Sleep Disorder, Adjustment[Title/Abstract] OR Environmental Sleep Disorder[Title/Abstract] OR Environmental Sleep Disorders[Title/Abstract] OR Sleep Disorders, Environmental[Title/Abstract] OR Sleep Disorder, Environmental[Title/Abstract] OR Limit-Setting Sleep Disorder[Title/Abstract] OR Limit Setting Sleep Disorder[Title/Abstract] OR Limit-Setting Sleep Disorders[Title/Abstract] OR Sleep Disorders, Limit-Setting[Title/Abstract] OR Sleep Disorder, Limit-Setting[Title/Abstract] OR Sleep Disorder, Limit Setting[Title/Abstract] OR Nocturnal Eating-Drinking Syndrome[Title/Abstract] OR Eating-Drinking Syndromes, Nocturnal[Title/Abstract] OR Eating-Drinking Syndrome, Nocturnal[Title/Abstract] OR Nocturnal Eating Drinking Syndrome[Title/Abstract] OR Nocturnal Eating-Drinking Syndromes[Title/Abstract] OR Syndrome, Nocturnal Eating-Drinking[Title/Abstract] OR Syndromes, Nocturnal Eating-Drinking[Title/Abstract] OR Dyssomnias[Title/Abstract] |
| 3 | ("Dyssomnias"[Mesh]) OR (sleep problems[Title/Abstract] OR sleep disturbances[Title/Abstract] OR sleep disorders[Title/Abstract] OR Dyssomnia[Title/Abstract] OR Sleep Disorders, Extrinsic[Title/Abstract] OR Extrinsic Sleep Disorder[Title/Abstract] OR Extrinsic Sleep Disorders[Title/Abstract] OR Sleep Disorder, Extrinsic[Title/Abstract] OR Adjustment Sleep Disorder[Title/Abstract] OR Adjustment Sleep Disorders[Title/Abstract] OR Sleep Disorders, Adjustment[Title/Abstract] OR Sleep Disorder, Adjustment[Title/Abstract] OR Environmental Sleep Disorder[Title/Abstract] OR Environmental Sleep Disorders[Title/Abstract] OR Sleep Disorders, Environmental[Title/Abstract] OR Sleep Disorder, Environmental[Title/Abstract] OR Limit-Setting Sleep Disorder[Title/Abstract] OR Limit Setting Sleep Disorder[Title/Abstract] OR Limit-Setting Sleep Disorders[Title/Abstract] OR Sleep Disorders, Limit-Setting[Title/Abstract] OR Sleep Disorder, Limit-Setting[Title/Abstract] OR Sleep Disorder, Limit Setting[Title/Abstract] OR Nocturnal Eating-Drinking Syndrome[Title/Abstract] OR Eating-Drinking Syndromes, Nocturnal[Title/Abstract] OR Eating-Drinking Syndrome, Nocturnal[Title/Abstract] OR Nocturnal Eating Drinking Syndrome[Title/Abstract] OR Nocturnal Eating-Drinking Syndromes[Title/Abstract] OR Syndrome, Nocturnal Eating-Drinking[Title/Abstract] OR Syndromes, Nocturnal Eating-Drinking[Title/Abstract] OR Dyssomnias[Title/Abstract]) |
| 4 | "Sleep"[Mesh] |
| 5 | Sleeping Habits[Title/Abstract] OR Sleep Habits[Title/Abstract] OR Habit, Sleep[Title/Abstract] OR Habits, Sleep[Title/Abstract] OR Sleep Habit[Title/Abstract] OR Sleeping Habit[Title/Abstract] OR Habit, Sleeping[Title/Abstract] OR Habits, Sleeping[Title/Abstract] OR sleep[Title/Abstract] |
| 6 | ("Sleep"[Mesh]) OR (Sleeping Habits[Title/Abstract] OR Sleep Habits[Title/Abstract] OR Habit, Sleep[Title/Abstract] OR Habits, Sleep[Title/Abstract] OR Sleep Habit[Title/Abstract] OR Sleeping Habit[Title/Abstract] OR Habit, Sleeping[Title/Abstract] OR Habits, Sleeping[Title/Abstract] OR sleep[Title/Abstract]) |
| 7 | "Depression"[Mesh] |
| 8 | Depressive Symptoms[Title/Abstract] OR Depressive Symptom[Title/Abstract] OR Symptom, Depressive[Title/Abstract] OR Emotional Depression[Title/Abstract] OR Depression, Emotional[Title/Abstract] OR depression[Title/Abstract] |
| 9 | ("Depression"[Mesh]) OR (Depressive Symptoms[Title/Abstract] OR Depressive Symptom[Title/Abstract] OR Symptom, Depressive[Title/Abstract] OR Emotional Depression[Title/Abstract] OR Depression, Emotional[Title/Abstract] OR depression[Title/Abstract]) |
| 10 | (("Dyssomnias"[Mesh]) OR (sleep problems[Title/Abstract] OR sleep disturbances[Title/Abstract] OR sleep disorders[Title/Abstract] OR Dyssomnia[Title/Abstract] OR Sleep Disorders, Extrinsic[Title/Abstract] OR Extrinsic Sleep Disorder[Title/Abstract] OR Extrinsic Sleep Disorders[Title/Abstract] OR Sleep Disorder, Extrinsic[Title/Abstract] OR Adjustment Sleep Disorder[Title/Abstract] OR Adjustment Sleep Disorders[Title/Abstract] OR Sleep Disorders, Adjustment[Title/Abstract] OR Sleep Disorder, Adjustment[Title/Abstract] OR Environmental Sleep Disorder[Title/Abstract] OR Environmental Sleep Disorders[Title/Abstract] OR Sleep Disorders, Environmental[Title/Abstract] OR Sleep Disorder, Environmental[Title/Abstract] OR Limit-Setting Sleep Disorder[Title/Abstract] OR Limit Setting Sleep Disorder[Title/Abstract] OR Limit-Setting Sleep Disorders[Title/Abstract] OR Sleep Disorders, Limit-Setting[Title/Abstract] OR Sleep Disorder, Limit-Setting[Title/Abstract] OR Sleep Disorder, Limit Setting[Title/Abstract] OR Nocturnal Eating-Drinking Syndrome[Title/Abstract] OR Eating-Drinking Syndromes, Nocturnal[Title/Abstract] OR Eating-Drinking Syndrome, Nocturnal[Title/Abstract] OR Nocturnal Eating Drinking Syndrome[Title/Abstract] OR Nocturnal Eating-Drinking Syndromes[Title/Abstract] OR Syndrome, Nocturnal Eating-Drinking[Title/Abstract] OR Syndromes, Nocturnal Eating-Drinking[Title/Abstract] OR Dyssomnias[Title/Abstract])) OR (("Sleep"[Mesh]) OR (Sleeping Habits[Title/Abstract] OR Sleep Habits[Title/Abstract] OR Habit, Sleep[Title/Abstract] OR Habits, Sleep[Title/Abstract] OR Sleep Habit[Title/Abstract] OR Sleeping Habit[Title/Abstract] OR Habit, Sleeping[Title/Abstract] OR Habits, Sleeping[Title/Abstract] OR sleep[Title/Abstract])) |
| 11 | (("Depression"[Mesh]) OR (Depressive Symptoms[Title/Abstract] OR Depressive Symptom[Title/Abstract] OR Symptom, Depressive[Title/Abstract] OR Emotional Depression[Title/Abstract] OR Depression, Emotional[Title/Abstract] OR depression[Title/Abstract])) AND (((("Dyssomnias"[Mesh]) OR (sleep problems[Title/Abstract] OR sleep disturbances[Title/Abstract] OR sleep disorders[Title/Abstract] OR Dyssomnia[Title/Abstract] OR Sleep Disorders, Extrinsic[Title/Abstract] OR Extrinsic Sleep Disorder[Title/Abstract] OR Extrinsic Sleep Disorders[Title/Abstract] OR Sleep Disorder, Extrinsic[Title/Abstract] OR Adjustment Sleep Disorder[Title/Abstract] OR Adjustment Sleep Disorders[Title/Abstract] OR Sleep Disorders, Adjustment[Title/Abstract] OR Sleep Disorder, Adjustment[Title/Abstract] OR Environmental Sleep Disorder[Title/Abstract] OR Environmental Sleep Disorders[Title/Abstract] OR Sleep Disorders, Environmental[Title/Abstract] OR Sleep Disorder, Environmental[Title/Abstract] OR Limit-Setting Sleep Disorder[Title/Abstract] OR Limit Setting Sleep Disorder[Title/Abstract] OR Limit-Setting Sleep Disorders[Title/Abstract] OR Sleep Disorders, Limit-Setting[Title/Abstract] OR Sleep Disorder, Limit-Setting[Title/Abstract] OR Sleep Disorder, Limit Setting[Title/Abstract] OR Nocturnal Eating-Drinking Syndrome[Title/Abstract] OR Eating-Drinking Syndromes, Nocturnal[Title/Abstract] OR Eating-Drinking Syndrome, Nocturnal[Title/Abstract] OR Nocturnal Eating Drinking Syndrome[Title/Abstract] OR Nocturnal Eating-Drinking Syndromes[Title/Abstract] OR Syndrome, Nocturnal Eating-Drinking[Title/Abstract] OR Syndromes, Nocturnal Eating-Drinking[Title/Abstract] OR Dyssomnias[Title/Abstract])) OR (("Sleep"[Mesh]) OR (Sleeping Habits[Title/Abstract] OR Sleep Habits[Title/Abstract] OR Habit, Sleep[Title/Abstract] OR Habits, Sleep[Title/Abstract] OR Sleep Habit[Title/Abstract] OR Sleeping Habit[Title/Abstract] OR Habit, Sleeping[Title/Abstract] OR Habits, Sleeping[Title/Abstract] OR sleep[Title/Abstract]))) |
| 12 | "Trazodone"[Mesh] |
| 13 | Tradozone[Title/Abstract] OR Trazodone Hydrochloride[Title/Abstract] OR AF1161[Title/Abstract] OR AF-1161[Title/Abstract] OR AF 1161[Title/Abstract] OR Molipaxin[Title/Abstract] OR Apo-Trazodone[Title/Abstract] OR Apo Trazodone[Title/Abstract] OR Deprax[Title/Abstract] OR Desyrel[Title/Abstract] OR Gen-Trazodone[Title/Abstract] OR Gen Trazodone[Title/Abstract] OR Novo-Trazodone[Title/Abstract] OR Novo Trazodone[Title/Abstract] OR Nu-Trazodone[Title/Abstract] OR Nu Trazodone[Title/Abstract] OR PMS-Trazodone[Title/Abstract] OR PMS Trazodone[Title/Abstract] OR Ratio-Trazodone[Title/Abstract] OR RatioTrazodone[Title/Abstract] OR Ratio Trazodone[Title/Abstract] OR Thombran[Title/Abstract] OR Trazodon Hexal[Title/Abstract] OR Trazodon-Neuraxpharm[Title/Abstract] OR TrazodonNeuraxpharm[Title/Abstract] OR Trazodon Neuraxpharm[Title/Abstract] OR Trittico[Title/Abstract] OR Trazon[Title/Abstract] OR Trazodone[Title/Abstract] |
| 14 | ("Trazodone"[Mesh]) OR (Tradozone[Title/Abstract] OR Trazodone Hydrochloride[Title/Abstract] OR AF1161[Title/Abstract] OR AF-1161[Title/Abstract] OR AF 1161[Title/Abstract] OR Molipaxin[Title/Abstract] OR Apo-Trazodone[Title/Abstract] OR Apo Trazodone[Title/Abstract] OR Deprax[Title/Abstract] OR Desyrel[Title/Abstract] OR Gen-Trazodone[Title/Abstract] OR Gen Trazodone[Title/Abstract] OR Novo-Trazodone[Title/Abstract] OR Novo Trazodone[Title/Abstract] OR Nu-Trazodone[Title/Abstract] OR Nu Trazodone[Title/Abstract] OR PMS-Trazodone[Title/Abstract] OR PMS Trazodone[Title/Abstract] OR Ratio-Trazodone[Title/Abstract] OR RatioTrazodone[Title/Abstract] OR Ratio Trazodone[Title/Abstract] OR Thombran[Title/Abstract] OR Trazodon Hexal[Title/Abstract] OR Trazodon-Neuraxpharm[Title/Abstract] OR TrazodonNeuraxpharm[Title/Abstract] OR Trazodon Neuraxpharm[Title/Abstract] OR Trittico[Title/Abstract] OR Trazon[Title/Abstract] OR Trazodone[Title/Abstract]) |
| 15 | (randomized controlled trial[Publication Type]) OR (randomized[Title/Abstract]) OR (placebo[Title/Abstract]) |
| 16 | (((("Depression"[Mesh]) OR (Depressive Symptoms[Title/Abstract] OR Depressive Symptom[Title/Abstract] OR Symptom, Depressive[Title/Abstract] OR Emotional Depression[Title/Abstract] OR Depression, Emotional[Title/Abstract] OR depression[Title/Abstract])) AND (((("Dyssomnias"[Mesh]) OR (sleep problems[Title/Abstract] OR sleep disturbances[Title/Abstract] OR sleep disorders[Title/Abstract] OR Dyssomnia[Title/Abstract] OR Sleep Disorders, Extrinsic[Title/Abstract] OR Extrinsic Sleep Disorder[Title/Abstract] OR Extrinsic Sleep Disorders[Title/Abstract] OR Sleep Disorder, Extrinsic[Title/Abstract] OR Adjustment Sleep Disorder[Title/Abstract] OR Adjustment Sleep Disorders[Title/Abstract] OR Sleep Disorders, Adjustment[Title/Abstract] OR Sleep Disorder, Adjustment[Title/Abstract] OR Environmental Sleep Disorder[Title/Abstract] OR Environmental Sleep Disorders[Title/Abstract] OR Sleep Disorders, Environmental[Title/Abstract] OR Sleep Disorder, Environmental[Title/Abstract] OR Limit-Setting Sleep Disorder[Title/Abstract] OR Limit Setting Sleep Disorder[Title/Abstract] OR Limit-Setting Sleep Disorders[Title/Abstract] OR Sleep Disorders, Limit-Setting[Title/Abstract] OR Sleep Disorder, Limit-Setting[Title/Abstract] OR Sleep Disorder, Limit Setting[Title/Abstract] OR Nocturnal Eating-Drinking Syndrome[Title/Abstract] OR Eating-Drinking Syndromes, Nocturnal[Title/Abstract] OR Eating-Drinking Syndrome, Nocturnal[Title/Abstract] OR Nocturnal Eating Drinking Syndrome[Title/Abstract] OR Nocturnal Eating-Drinking Syndromes[Title/Abstract] OR Syndrome, Nocturnal Eating-Drinking[Title/Abstract] OR Syndromes, Nocturnal Eating-Drinking[Title/Abstract] OR Dyssomnias[Title/Abstract])) OR (("Sleep"[Mesh]) OR (Sleeping Habits[Title/Abstract] OR Sleep Habits[Title/Abstract] OR Habit, Sleep[Title/Abstract] OR Habits, Sleep[Title/Abstract] OR Sleep Habit[Title/Abstract] OR Sleeping Habit[Title/Abstract] OR Habit, Sleeping[Title/Abstract] OR Habits, Sleeping[Title/Abstract] OR sleep[Title/Abstract])))) AND (("Trazodone"[Mesh]) OR (Tradozone[Title/Abstract] OR Trazodone Hydrochloride[Title/Abstract] OR AF1161[Title/Abstract] OR AF-1161[Title/Abstract] OR AF 1161[Title/Abstract] OR Molipaxin[Title/Abstract] OR Apo-Trazodone[Title/Abstract] OR Apo Trazodone[Title/Abstract] OR Deprax[Title/Abstract] OR Desyrel[Title/Abstract] OR Gen-Trazodone[Title/Abstract] OR Gen Trazodone[Title/Abstract] OR Novo-Trazodone[Title/Abstract] OR Novo Trazodone[Title/Abstract] OR Nu-Trazodone[Title/Abstract] OR Nu Trazodone[Title/Abstract] OR PMS-Trazodone[Title/Abstract] OR PMS Trazodone[Title/Abstract] OR Ratio-Trazodone[Title/Abstract] OR RatioTrazodone[Title/Abstract] OR Ratio Trazodone[Title/Abstract] OR Thombran[Title/Abstract] OR Trazodon Hexal[Title/Abstract] OR Trazodon-Neuraxpharm[Title/Abstract] OR TrazodonNeuraxpharm[Title/Abstract] OR Trazodon Neuraxpharm[Title/Abstract] OR Trittico[Title/Abstract] OR Trazon[Title/Abstract] OR Trazodone[Title/Abstract]))) AND ((randomized controlled trial[Publication Type]) OR (randomized[Title/Abstract]) OR (placebo[Title/Abstract])) |

Supplementary Table S2. Additional PubMed search strategy for polysomnography-related studies

| **Search number** | **Query** |
| --- | --- |
| 1 | ("Trazodone"[Mesh]) OR (Tradozone[Title/Abstract] OR Trazodone Hydrochloride[Title/Abstract] OR AF1161[Title/Abstract] OR AF-1161[Title/Abstract] OR AF 1161[Title/Abstract] OR Molipaxin[Title/Abstract] OR Apo-Trazodone[Title/Abstract] OR Apo Trazodone[Title/Abstract] OR Deprax[Title/Abstract] OR Desyrel[Title/Abstract] OR Gen-Trazodone[Title/Abstract] OR Gen Trazodone[Title/Abstract] OR Novo-Trazodone[Title/Abstract] OR Novo Trazodone[Title/Abstract] OR Nu-Trazodone[Title/Abstract] OR Nu Trazodone[Title/Abstract] OR PMS-Trazodone[Title/Abstract] OR PMS Trazodone[Title/Abstract] OR Ratio-Trazodone[Title/Abstract] OR RatioTrazodone[Title/Abstract] OR Ratio Trazodone[Title/Abstract] OR Thombran[Title/Abstract] OR Trazodon Hexal[Title/Abstract] OR Trazodon-Neuraxpharm[Title/Abstract] OR TrazodonNeuraxpharm[Title/Abstract] OR Trazodon Neuraxpharm[Title/Abstract] OR Trittico[Title/Abstract] OR Trazon[Title/Abstract] OR Trazodone[Title/Abstract]) |
| 2 | ("Depression"[Mesh]) OR (Depressive Symptoms[Title/Abstract] OR Depressive Symptom[Title/Abstract] OR Symptom, Depressive[Title/Abstract] OR Emotional Depression[Title/Abstract] OR Depression, Emotional[Title/Abstract] OR depression[Title/Abstract]) |
| 3 | (("Sleep"[Mesh]) OR (Sleeping Habits[Title/Abstract] OR Sleep Habits[Title/Abstract] OR Habit, Sleep[Title/Abstract] OR Habits, Sleep[Title/Abstract] OR Sleep Habit[Title/Abstract] OR Sleeping Habit[Title/Abstract] OR Habit, Sleeping[Title/Abstract] OR Habits, Sleeping[Title/Abstract] OR sleep[Title/Abstract])) OR (("Dyssomnias"[Mesh]) OR (sleep problems[Title/Abstract] OR sleep disturbances[Title/Abstract] OR sleep disorders[Title/Abstract] OR Dyssomnia[Title/Abstract] OR Sleep Disorders, Extrinsic[Title/Abstract] OR Extrinsic Sleep Disorder[Title/Abstract] OR Extrinsic Sleep Disorders[Title/Abstract] OR Sleep Disorder, Extrinsic[Title/Abstract] OR Adjustment Sleep Disorder[Title/Abstract] OR Adjustment Sleep Disorders[Title/Abstract] OR Sleep Disorders, Adjustment[Title/Abstract] OR Sleep Disorder, Adjustment[Title/Abstract] OR Environmental Sleep Disorder[Title/Abstract] OR Environmental Sleep Disorders[Title/Abstract] OR Sleep Disorders, Environmental[Title/Abstract] OR Sleep Disorder, Environmental[Title/Abstract] OR Limit-Setting Sleep Disorder[Title/Abstract] OR Limit Setting Sleep Disorder[Title/Abstract] OR Limit-Setting Sleep Disorders[Title/Abstract] OR Sleep Disorders, Limit-Setting[Title/Abstract] OR Sleep Disorder, Limit-Setting[Title/Abstract] OR Sleep Disorder, Limit Setting[Title/Abstract] OR Nocturnal Eating-Drinking Syndrome[Title/Abstract] OR Eating-Drinking Syndromes, Nocturnal[Title/Abstract] OR Eating-Drinking Syndrome, Nocturnal[Title/Abstract] OR Nocturnal Eating Drinking Syndrome[Title/Abstract] OR Nocturnal Eating-Drinking Syndromes[Title/Abstract] OR Syndrome, Nocturnal Eating-Drinking[Title/Abstract] OR Syndromes, Nocturnal Eating-Drinking[Title/Abstract] OR Dyssomnias[Title/Abstract])) |
| 4 | "Polysomnography"[Mesh] |
| 6 | Polysomnographies OR Monitoring, Sleep OR Sleep Monitoring OR Somnography OR Somnographies |
| 7 | ("Polysomnography"[Mesh]) OR (Polysomnographies OR Monitoring, Sleep OR Sleep Monitoring OR Somnography OR Somnographies) |
| 8 | (((("Polysomnography"[Mesh]) OR (Polysomnographies OR Monitoring, Sleep OR Sleep Monitoring OR Somnography OR Somnographies)) AND ((("Sleep"[Mesh]) OR (Sleeping Habits[Title/Abstract] OR Sleep Habits[Title/Abstract] OR Habit, Sleep[Title/Abstract] OR Habits, Sleep[Title/Abstract] OR Sleep Habit[Title/Abstract] OR Sleeping Habit[Title/Abstract] OR Habit, Sleeping[Title/Abstract] OR Habits, Sleeping[Title/Abstract] OR sleep[Title/Abstract])) OR (("Dyssomnias"[Mesh]) OR (sleep problems[Title/Abstract] OR sleep disturbances[Title/Abstract] OR sleep disorders[Title/Abstract] OR Dyssomnia[Title/Abstract] OR Sleep Disorders, Extrinsic[Title/Abstract] OR Extrinsic Sleep Disorder[Title/Abstract] OR Extrinsic Sleep Disorders[Title/Abstract] OR Sleep Disorder, Extrinsic[Title/Abstract] OR Adjustment Sleep Disorder[Title/Abstract] OR Adjustment Sleep Disorders[Title/Abstract] OR Sleep Disorders, Adjustment[Title/Abstract] OR Sleep Disorder, Adjustment[Title/Abstract] OR Environmental Sleep Disorder[Title/Abstract] OR Environmental Sleep Disorders[Title/Abstract] OR Sleep Disorders, Environmental[Title/Abstract] OR Sleep Disorder, Environmental[Title/Abstract] OR Limit-Setting Sleep Disorder[Title/Abstract] OR Limit Setting Sleep Disorder[Title/Abstract] OR Limit-Setting Sleep Disorders[Title/Abstract] OR Sleep Disorders, Limit-Setting[Title/Abstract] OR Sleep Disorder, Limit-Setting[Title/Abstract] OR Sleep Disorder, Limit Setting[Title/Abstract] OR Nocturnal Eating-Drinking Syndrome[Title/Abstract] OR Eating-Drinking Syndromes, Nocturnal[Title/Abstract] OR Eating-Drinking Syndrome, Nocturnal[Title/Abstract] OR Nocturnal Eating Drinking Syndrome[Title/Abstract] OR Nocturnal Eating-Drinking Syndromes[Title/Abstract] OR Syndrome, Nocturnal Eating-Drinking[Title/Abstract] OR Syndromes, Nocturnal Eating-Drinking[Title/Abstract] OR Dyssomnias[Title/Abstract])))) AND (("Depression"[Mesh]) OR (Depressive Symptoms[Title/Abstract] OR Depressive Symptom[Title/Abstract] OR Symptom, Depressive[Title/Abstract] OR Emotional Depression[Title/Abstract] OR Depression, Emotional[Title/Abstract] OR depression[Title/Abstract]))) AND (("Trazodone"[Mesh]) OR (Tradozone[Title/Abstract] OR Trazodone Hydrochloride[Title/Abstract] OR AF1161[Title/Abstract] OR AF-1161[Title/Abstract] OR AF 1161[Title/Abstract] OR Molipaxin[Title/Abstract] OR Apo-Trazodone[Title/Abstract] OR Apo Trazodone[Title/Abstract] OR Deprax[Title/Abstract] OR Desyrel[Title/Abstract] OR Gen-Trazodone[Title/Abstract] OR Gen Trazodone[Title/Abstract] OR Novo-Trazodone[Title/Abstract] OR Novo Trazodone[Title/Abstract] OR Nu-Trazodone[Title/Abstract] OR Nu Trazodone[Title/Abstract] OR PMS-Trazodone[Title/Abstract] OR PMS Trazodone[Title/Abstract] OR Ratio-Trazodone[Title/Abstract] OR RatioTrazodone[Title/Abstract] OR Ratio Trazodone[Title/Abstract] OR Thombran[Title/Abstract] OR Trazodon Hexal[Title/Abstract] OR Trazodon-Neuraxpharm[Title/Abstract] OR TrazodonNeuraxpharm[Title/Abstract] OR Trazodon Neuraxpharm[Title/Abstract] OR Trittico[Title/Abstract] OR Trazon[Title/Abstract] OR Trazodone[Title/Abstract])) |

Supplementary Table S3. Characteristics of the eight included studies.

| First author | Year | Country | Sample size | Age，  mean±SD(range) | Female% | Diagnostic  Criteria | Type of Study | Intervention  (Experimental/  Control) | Dosage | Duration | Concurrent  therapy | Outcome  measures |
| --- | --- | --- | --- | --- | --- | --- | --- | --- | --- | --- | --- | --- |
| Bavato | 2025 | Switzerland | 23 | 26.8±6.5  (20~45 ) | 69.57 | DSM-V | single-center,  Randomized, double-blind, placebo-and active  comparator-controlled, crossover trial | Experimental: Trazodone  /Control: Placebo | 1.5mg/kg  (72–156 mg) | one night | Stable antidepressant treatment | PSG(TST,SL,WASO,N1(%),N2(%),REM(%),SE),  ≥1AE,  Dizziness, Somnolence, Nausea |
| Zhang | 2014 | China | 183  (Experimental)/  180  (Control) | 39.5±12.7(Experim-  ental)/  38.3±12.2(Control)  (range:NR) | 61.43 | DSM-IV | Multicenter, randomized, double-blind, placebo-controlled trial | Experimental: Prolonged-release trazodone /Control: Placebo | 150–450 mg/day | six weeks | None | HAMD, Response rate, CGI-I  Response rate,  ≥1 AE, Dizziness,  Dry mouth, Somnolence, Nausea |
| Sheehan | 2009 | The USA,  Canada. | 202  (Experimental)/  204  (Control) | 43.9±13.1  (NR) | 64.04 | DSM-IV | Multicenter, randomized, double-blind, placebo-controlled trial | Experimental: Extended-release trazodone / Control: Placebo | 150–375 mg/day | six weeks | None | HAMD, Response rate, CGI-I  response rate, ≥1 AE,  the discontinuation rate due to AEs, Dizziness, Dry mouth,  Somnolence, Nausea |
| Kaynak | 2004 | Turkey | 12 | 42±9  (30~59) | 100 | DSM-IV | randomized,  double-blind, placebo-controlled,  Crossover trial | Experimental: Trazodone/ Control: Placebo | 100  mg/day | one week | SSRIs | HAMD,PSG(TST,SL,SE,N1(%),N2(%),SWS(%),REM(%),REM-L),≥1 AE |
| Arriaga | 1997 | Portugal | 9 | 43.33±7.37  (32~55) | 100 | DSM-IV | Single-blind, within-subject, placebo-controlled trial | Trazodone | 50-250  mg/day | five weeks | None | PSG(TST,SE,SL,REM-L,N1(%),N2(%),SWS(%),REM(%)) |
| Parrino | 1994 | Italy | 6 | 46.5±4.9  (40~55) | 50 | DSM-III | Single-blind, within-subject, placebo-controlled trial | trazodone controlled release | 50-150  mg/day | six weeks | None | PSG(TST,SE,SL,REM-L,WASO,N1(%),N2(%),SWS(%),REM(%)) |
| Cunningham | 1994 | The USA,  Canada. | 77  (Experimental)/  76  (Control) | 40.7±NR  (NR) | 33.3 | DSM-III | Multicenter,  randomized, double-blind, placebo-controlled trial | Experimental: Trazodone/ Control: Placebo | 150–400 mg/day | six weeks | None | the discontinuation rate due to AEs, Dizziness, Dry mouth,  Somnolence, Nausea |
| van Bemmel | 1992 | The Netherlands | 8 | 43.5±7.3  (34~56) | 75 | DSM-III | Single-blind, within-subject, placebo-controlled trial | Trazodone | 100–400 mg/day | five weeks | None | PSG(TST,SE,SL,REM-L,WASO,N1(%),N2(%),SWS(%),REM(%)) |

≥1 AE: proportion of patients who experienced at least one adverse event; CGI-I responders: patients assessed by investigators as “minimally improved”, “much improved”, or “very much improved” on the Clinical Global Impression-Improvement scale; DSM: Diagnostic and Statistical Manual of Mental Disorders; HAMD: Hamilton Depression Scale; N1: non-rapid eye movement stage 1; N2: non-rapid eye movement stage 2; NR: not reported; PSG: polysomnography; REM: rapid eye movement; REM-L: latency to rapid eye movement sleep; Response rate: percentage of patients achieving a ≥50% reduction in the total HAMD-17 score from baseline to the end of the study; SE: sleep efficiency index; SL: sleep latency; SSRIs: selective serotonin reuptake inhibitors; SWS: slow-wave sleep; TST: total sleep time; WASO: waking time after sleep onset.

Supplementary Table S4. Sensitivity analyses under different correlation coefficients (r = 0.2 and 0.8)

| Outcome | r | No. of studies | Heterogeneity | MD | 95% CI | Z | P-value |
| --- | --- | --- | --- | --- | --- | --- | --- |
| TST, min | 0.2 | 4 | P=0.007; I^2^=75.2% | 49.572 | 20.655, 78.489 | 3.36 | 0.001 |
|  | 0.8 | 4 | P=0.000; I^2^=89.2% | 51.311 | 26.764, 75.857 | 4.10 | 0.000 |
| SL, min | 0.2 | 4 | P=0.127; I^2^=47.4% | -14.295 | -23.021, -5.569 | -3.21 | 0.001 |
|  | 0.8 | 4 | P=0.000; I^2^=85.3% | -15.353 | -24.509, -6.198 | -3.29 | 0.001 |
| SE, % | 0.2 | 4 | P=0.607; I^2^=0.0% | 6.873 | 4.627,9.120 | 6.00 | 0.000 |
|  | 0.8 | 4 | P=0.141; I^2^=45.1% | 7.836 | 5.522,10.150 | 6.64 | 0.000 |
| REM-L, min | 0.2 | 4 | P=0.107; I^2^=50.9% | 16.083 | -16.131,48.297 | 0.98 | 0.328 |
|  | 0.8 | 4 | P=0.005; I^2^=76.8% | 16.129 | -18.930,51.188 | 0.90 | 0.367 |
| WASO, min | 0.2 | 2 | P=0.015; I^2^=83.3% | -31.783 | -64.901,1.334 | -1.88 | 0.06 |
|  | 0.8 | 2 | P=0.000; I^2^=93.5% | -32.234 | -65.356,0.888 | -1.91 | 0.056 |
| N1, % | 0.2 | 4 | P=0.001; I^2^=83.0% | -0.150 | -3.226,2.926 | -0.10 | 0.924 |
|  | 0.8 | 4 | P=0.000; I^2^=95.1% | 0.019 | -3.096,3.135 | 0.01 | 0.990 |
| N2, % | 0.2 | 4 | P=0.003; I^2^=78.8% | -9.214 | -16.712, -1.717 | -2.41 | 0.016 |
|  | 0.8 | 4 | P=0.000; I^2^=92.8% | -8.984 | -16.507, -1.461 | -2.34 | 0.019 |
| SWS, % | 0.2 | 4 | P=0.108; I^2^=50.6% | 8.130 | 3.567,12.693 | 3.49 | 0.000 |
|  | 0.8 | 4 | P=0.000; I^2^=84.3% | 7.999 | 3.170,12.829 | 3.25 | 0.001 |
| REM, % | 0.2 | 4 | P=0.034; I^2^=65.3% | -0.033 | -4.224,4.158 | -0.02 | 0.988 |
|  | 0.8 | 4 | P=0.000; I^2^=87.6% | 0.183 | -3.843,4.209 | 0.09 | 0.929 |

MD = mean difference; CI = confidence interval; I² = inconsistency statistic.

Supplementary Table S5. Sensitivity analyses of primary outcomes after exclusion of studies contributing substantially to heterogeneity

| Outcome | No. of studies | Heterogeneity | MD | 95%CI | Z | P-value |
| --- | --- | --- | --- | --- | --- | --- |
| TST, min | 3 | P=0.533; I^2^=0% | 30.840 | 22.786, 38.893 | 7.51 | 0.000 |
| SL，min | 3 | P=0.274; I^2^=22.8% | -10.611 | -17.444, -3.778 | 3.04 | 0.002 |
| REM-L, min | 3 | P=0.388; I^2^=0.0% | 6.429 | -13.454,26.312 | 0.63 | 0.526 |
| N2, % | 3 | P=0.175; I^2^=42.7% | -13.125 | -17.212, -9.039 | 6.30 | 0.000 |
| SWS, % | 3 | P=0.373; I^2^=0.0% | 9.995 | 7.018,12.972 | 6.58 | 0.000 |

The table presents the pooled effect estimates and heterogeneity after exclusion. Abbreviations: MD = mean difference; CI = confidence interval; I² = inconsistency statistic.

Supplementary Table S6. Leave-one-out sensitivity analysis for the proportion of N2 sleep.

| Study removed | I^2^ | MD | 95%CI | Z | P-value |
| --- | --- | --- | --- | --- | --- |
| Arriaga 1997 | 90.5% | -8.504 | -19.701,2.693 | 1.49 | 0.137 |
| Parrino 1994 | 77.8% | -6.434 | -14.549,1.682 | 1.55 | 0.120 |
| Van Bemmel 1992 | 42.7% | -13.125 | -17.212, -9.039 | 6.30 | 0.000 |
| Kaynak 2004 | 90.5% | -8.529 | -18.166,1.109 | 1.73 | 0.083 |

Each row represents the pooled effect estimates and heterogeneity after excluding the specified study. Abbreviations: MD = mean difference; CI = confidence interval; I² = inconsistency statistic.

Supplementary Table S7. Meta-analysis results for sleep architecture (RCTs only)

| Outcome | No. of studies | Heterogeneity | MD | 95%CI | Z | P-value |
| --- | --- | --- | --- | --- | --- | --- |
| TST, min | 2 | P=0.34; I^2^=0.0% | 26.68 | 5.03,48.33 | 2.42 | 0.02 |
| SL, min | 2 | P=0.21; I^2^=37% | 0.13 | -8.89,9.15 | 0.03 | 0.98 |
| SE, % | 2 | P=0.33; I^2^=0.0% | 6.09 | 1.42,10.75 | 2.56 | 0.01 |
| REM-L, min | 1 | - | -14 | -100.11,72.11 | 0.32 | 0.75 |
| WASO, min | 2 | P=0.34; I^2^=0.0% | -19.52 | -29.95，-9.09 | 3.67 | 0.0002 |
| N1, % | 2 | P=0.004; I^2^=88% | -1.39 | -6.77,4.00 | 0.50 | 0.61 |
| N2, % | 2 | P=0.24; I^2^=26% | -3.92 | -9.81,1.98 | 1.30 | 0.19 |
| SWS, % | 2 | P=0.14; I^2^=55% | 7.22 | -0.93,15.38 | 1.74 | 0.08 |
| REM, % | 2 | P=0.20; I^2^=38% | -1.88 | -5.40,1.54 | 1.05 | 0.29 |

Only RCTs were included in this supplementary analysis. Abbreviations: MD = mean difference; CI = confidence interval; I² = inconsistency statistic.
